# New Rural Focus of Plague, Algeria

**DOI:** 10.3201/eid1610.091854

**Published:** 2010-10

**Authors:** Idir Bitam, Saravanan Ayyadurai, Tahar Kernif, Mohammed Chetta, Nabil Boulaghman, Didier Raoult, Michel Drancourt

**Affiliations:** Author affiliations: Institut Pasteur d’Algérie, Hamma, Algeria (I. Bitam, T. Kernif);; Université de la Méditerranée, Marseille, France (S. Ayyadurai, D. Raoult, M. Drancourt);; Hôpital Universitaire de Laghouat, Laghouat, Algeria (M. Chetta, N. Boulaghman)

**Keywords:** Bacteria, plague, vector-borne infections, bubonic plague, Yersinia pestis, Orientalis plague, pneumonic plague, Algeria, letter

**To the Editor:** Plague is a deadly rodent-associated flea-borne zoonosis caused by the bacterium *Yersinia pestis* (*1*). Human plague periodically reemerges in so-called plague foci, as illustrated by the 2003 reemergence of human plague in the Oran area, Algeria (*2**,**3*). We report emergence of a new plague focus in a remote region of Algeria.

In July 2008, three patients came to Laghouat University Hospital with signs of severe infection and painful, inflamed, enlarged lymph nodes suggestive of buboes. One additional patient became ill with pneumonia and coma after a bubo appeared. The patients were nomads living in a 24-person camp in Thait El Maa in the Laghouat area, 550 km southwest of Algiers (Figure). Plague was confirmed by culturing *Y. pestis* from 1 bubo aspirate. Ten days of oral doxycycline (4 mg/kg/d) combined with oral rifampin (20 mg/kg/d) and intramuscular gentamicin (3 mg/kg/d) cured the patients with bubonic plague, but the patient with pneumonic plague died.

**Figure:**
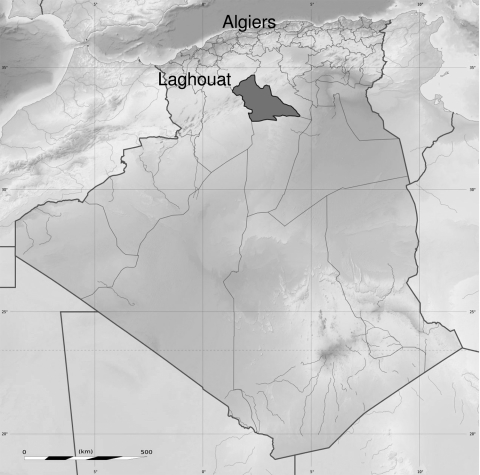
Location of a new rural plague focus in a nomad camp in Laghouat (dark gray shading; 35°29′N, 0°32′E), Algeria.

In January 2009, eight individuals of the rodent species *Meriones shawii* (Shaw’s jird) and 2 *Psamommys obesus* (fat sand rats) were trapped inside nomads’ tents (H.P. Sherman Traps, Tallahassee, FL, USA). At time of capture, there was a cold wind with blowing sand, and, after visual inspection of the rodents, efforts to recover fleas failed. DNA from the rodents’ spleens was extracted by using the QIAamp Tissue Kit (QIAGEN, Hilden, Germany) at the Medical Entomology Unit Laboratory, Pasteur Institute, Algiers, and subjected to PCR amplification of the plasminogen activator gene (*pla*) from 6 *M. shawii* jirds. Negative controls (DNA extracted from uninfected fleas maintained as colonies in Medical Entomology Unit Laboratory was used in the absence of negative animal tissue) remained negative.

After sequencing, the PCR amplicons showed 100% sequence identity with *Y. pestis* reference sequences. Identification was further confirmed in Marseille, France, by culturing 2 rodent glycerol-negative *Y. pestis* isolates (Algeria 1 and Algeria 2) and sequencing *pla*, *caf*, and *glp*D genes. The latter sequence was identical to the reference *Y. pestis* CO92, an Orientalis biotype. Multispacer sequence typing found the following combination: spacer Yp3, type 5; Yp4, 1; Yp5, 1; Yp7, 8; Yp8, 2; Yp9, 2; and Yp10, 1, a pattern that is typical for all Orientalis isolates investigated by this method but does not match the combinations observed for other genotypes. The original spacer Yp7 type 8 ruled out contamination (*4*).

National health records indicate that plague foci have been known for decades in Algiers, Kahelia, Aumale, Philippeville, and Oran, where plague reemerged in 2003 after its abence for >50 years (*2**,**3*). In the Oran outbreak, it was not clear if reemergence resulted from importation through the international port of Oran or from a previously unknown rural focus (*3*). The Laghouat area was not previously known as a plague focus, and plague must therefore be regarded as an emerging disease in this region.

No patients with plague reported handling sick animals. Thus, the patients likely acquired plague from rodent flea bites. Because human ectoparasites were not found on the nomads, rodent ectoparasites must have transmitted the disease. It is unlikely plague had been imported into this region; the 2 rodent species from which *Y. pestis* was recovered are present in the area. To the best of the nomads’ knowledge, there have been no reports of movements of commensal rats or other plague-susceptible rodents into the area near the sites where the patients acquired their illnesses.

We found *Y. pestis* in *M. shawii* jirds, a native rodent species living in close contact with human populations. *M. shawii* jirds have been shown to be a plague-resistant species (*5*) and thus are an efficient reservoir for *Y. pestis.* These data verify the presence of a new, rural zoonotic focus of plague. This situation is worrisome because nomads remain in close contact with rodents and fleas and the risk of further outbreaks remains high. In Oran and Laghouat, an Orientalis biotype sharing the same Yp8 and Yp9 spacer sequences was found, but limited multiple spacer typing of Oran strains hampered further comparisons beyond the biotype level (*3*).

A plague focus has been recently detected in a neighboring Libyan focus located at the same latitude as the Laghouat area (*6*). Our report suggests that extending surveillance to adjacent Libya and Mauritania, which also have natural foci of plague, is necessary. The reasons for emergence of plague in these regions are unknown, but *Y. pestis* can survive in the soil under laboratory conditions, possibly providing the opportunity for rodents to be infected and promoting reemergence of the disease (*7**,**8*).

Emergence of plague in an area of Algeria where it had never been reported illustrates the necessity to reinforce surveillance of plague in possible rodent hosts and their ectoparasites, which are in contact with humans, to prevent emergence and reemergence of this deadly infection. Surveillance should be maintained to monitor this natural focus and potential spread of plague that might occur because of climatic or habitat influences (*9*).
